# Supplementary material for: cdev: a ground-truth based measure to evaluate RNA-seq normalization performance
Source: PeerJ. 2021 Oct 4;9:e12233. doi: 10.7717/peerj.12233 (PMC8496462; doi:10.7717/peerj.12233)
Supplement: Supplemental Information 1 [file peerj-09-12233-s001.pdf]

cdev: A ground-truth based measure to evaluate RNA-seq normalization performance
Supplemental Information

Diem-Trang Tran, Matthew Might

August 29, 2021

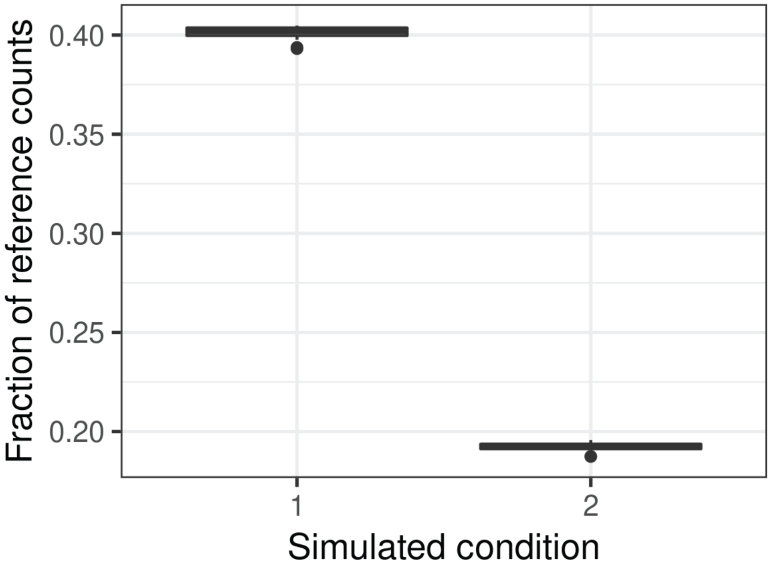

Fig. S1: **The proportion of true reference counts**, the simulation counterpart of the fraction of reads mapped to ERCC spike-ins.

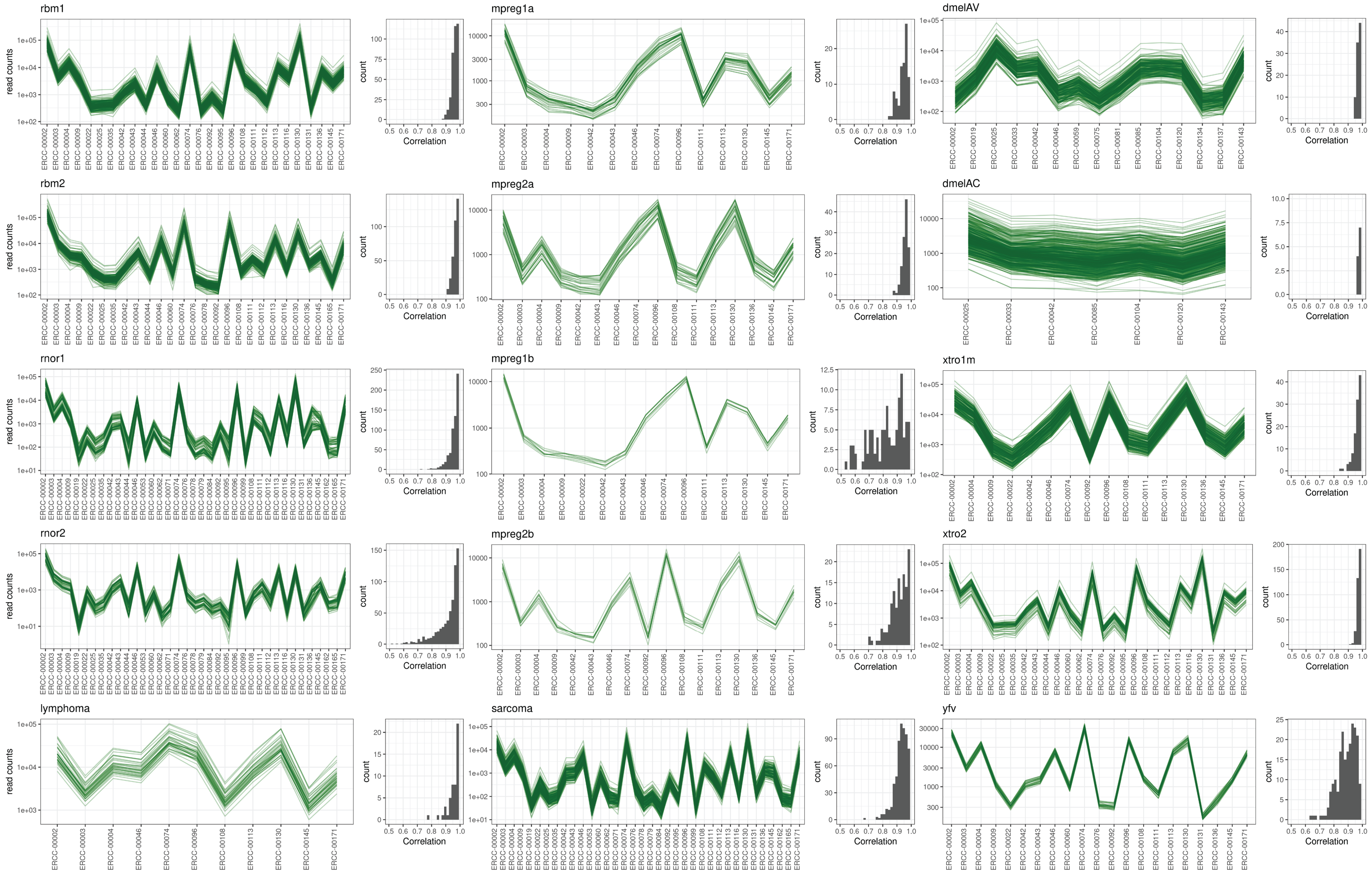

Fig. S2: Correlation of the ERCC spike-ins selected as true references, visualized in parallel coordinate plots (PCP) and histograms. In PCP, RNA species (ERCC spike-in) constitute the parallel y-axes and samples are plotted as polylines. Parallel lines indicates high correlation.

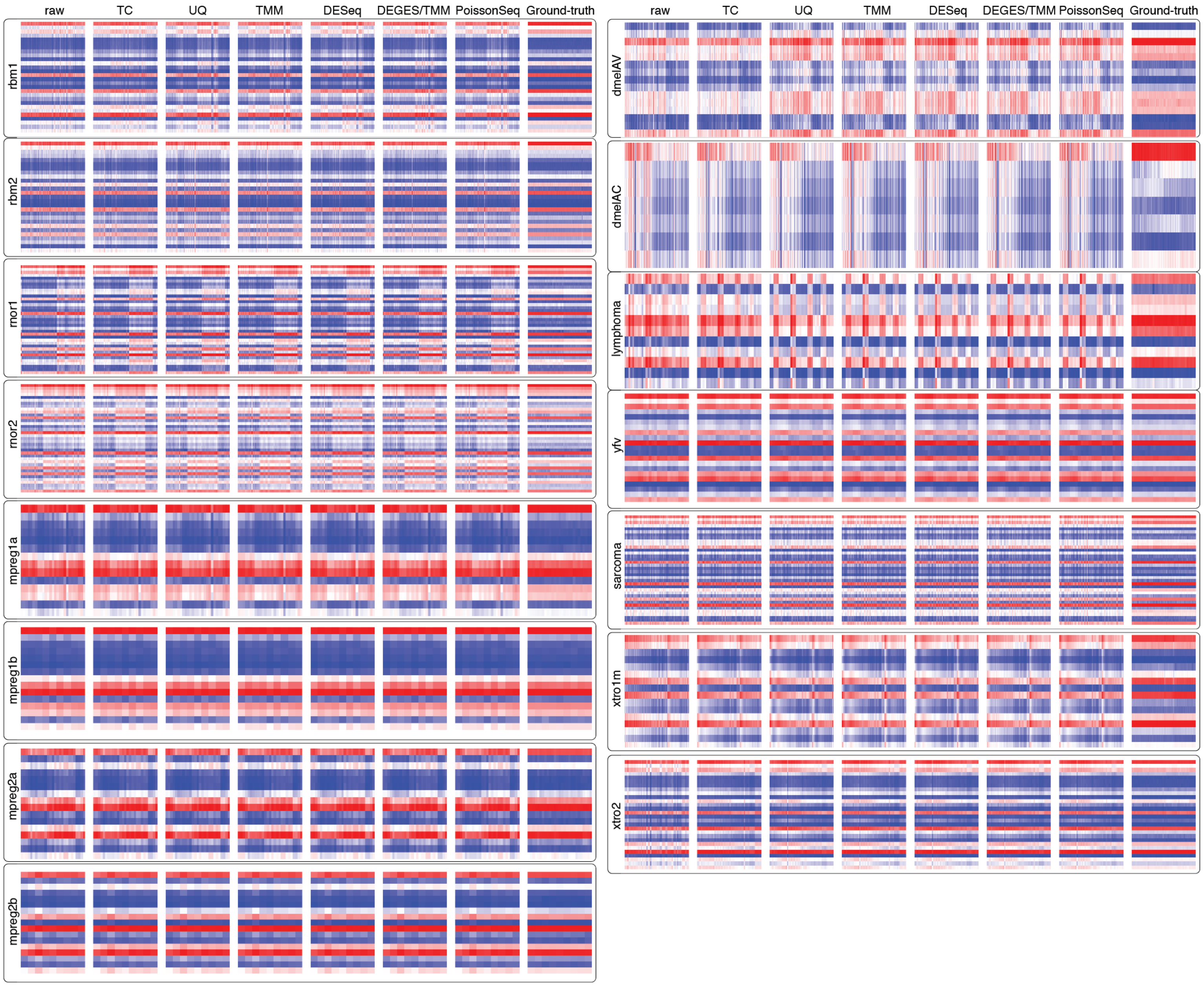

Fig. S3: **Expression levels of ERCC spike-ins selected as true references, after normalizing by various methods**, in comparison with no normalization (*raw*) and ground-truth normalization.

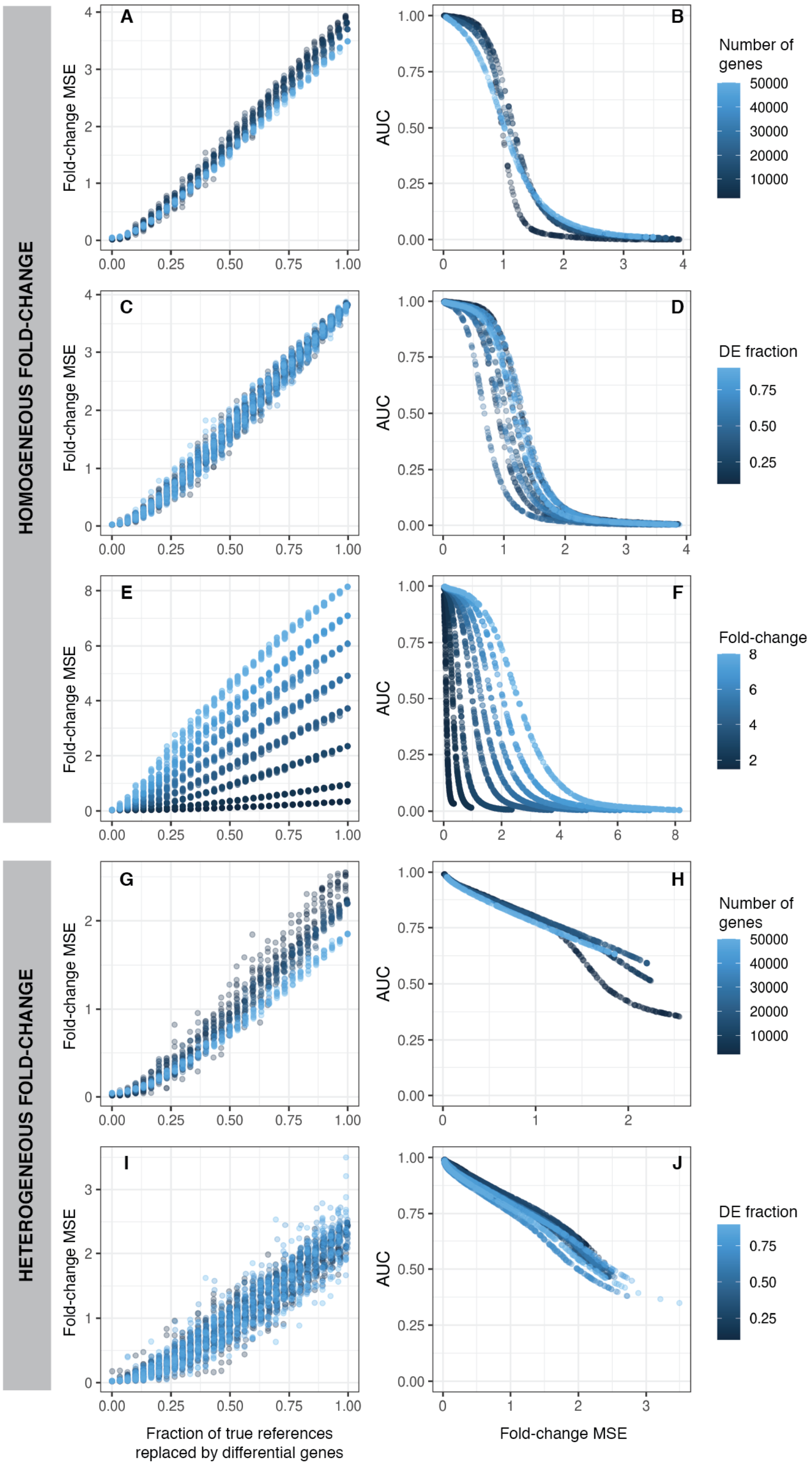

Fig. S4: **Fold-change MSE as a function of normalization quality on simulation data.** Left column: Fold-change MSE as the function of reference set quality, measured in the fraction of true differential genes. Right column: Fold-change MSE as a predictor of DE detection performance. Panels (**A-F**) represent the relationship on homogeneous fold-change simulation, panels (**G-J**) on heterogeneous fold-change simulation.

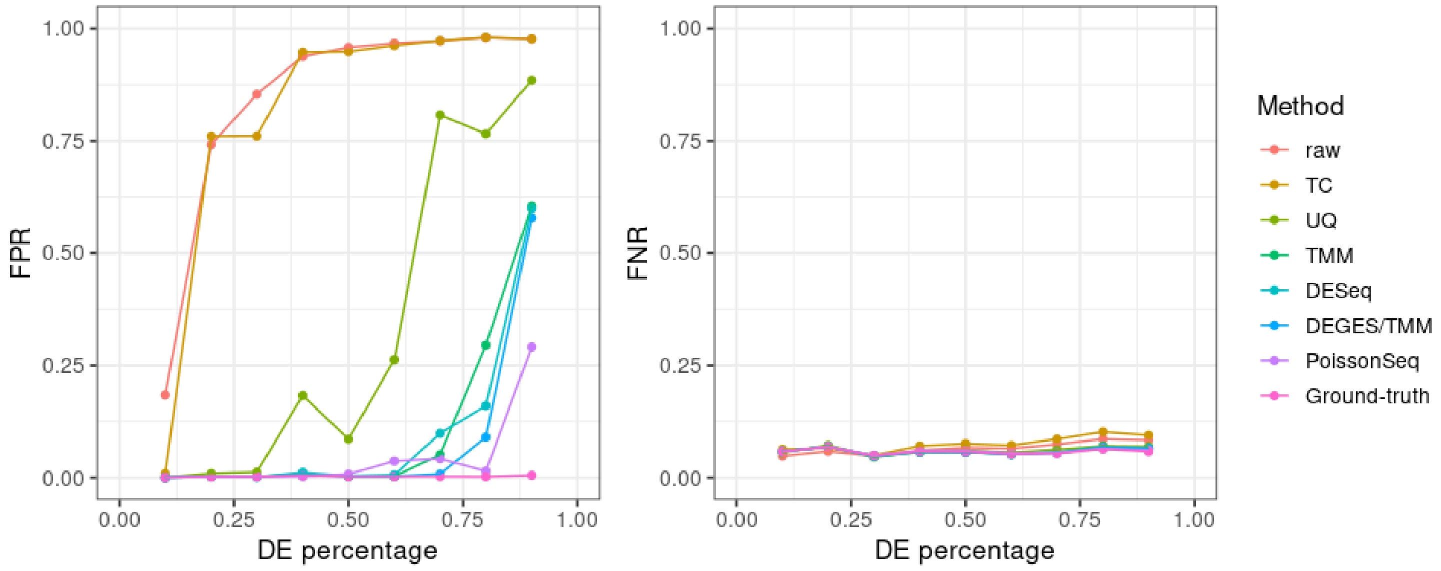

Fig. S5: **False positive rate (FPR) and False negative rate (FNR) of DE detection on simulated data sets normalized by different methods.**

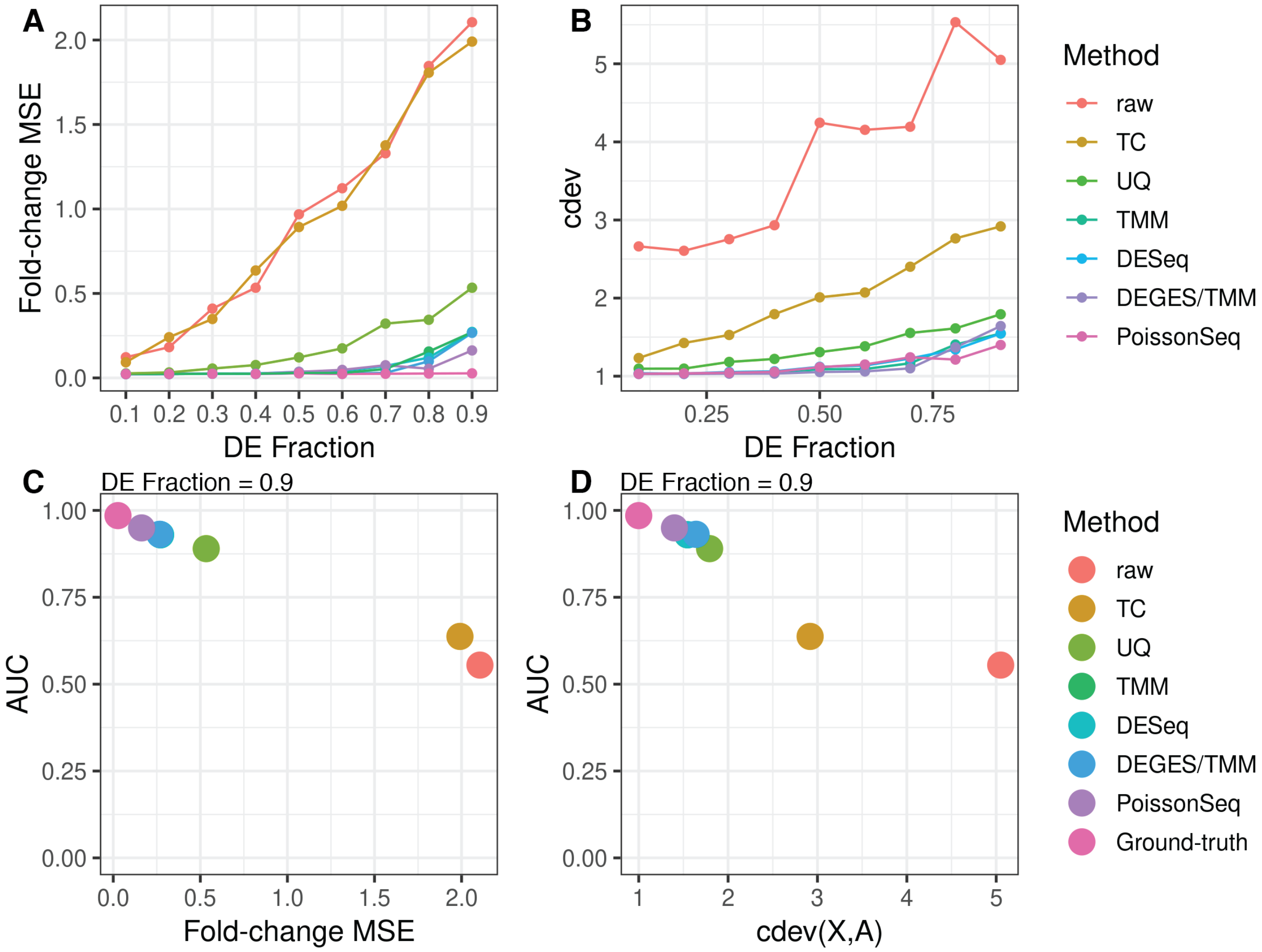

Fig. S6: **Fold-change MSE and *cdev* in comparing common normalizers on simulation data sets**. (A,B) Performance of various normalizers, measured in fold-change MSE and *cdev*, on data sets simulated at increasing fraction of differential expression. (C,D) AUC of DE detection as a function of fold-change MSE and *cdev*, on the simulation with 90% of the genes being differential.

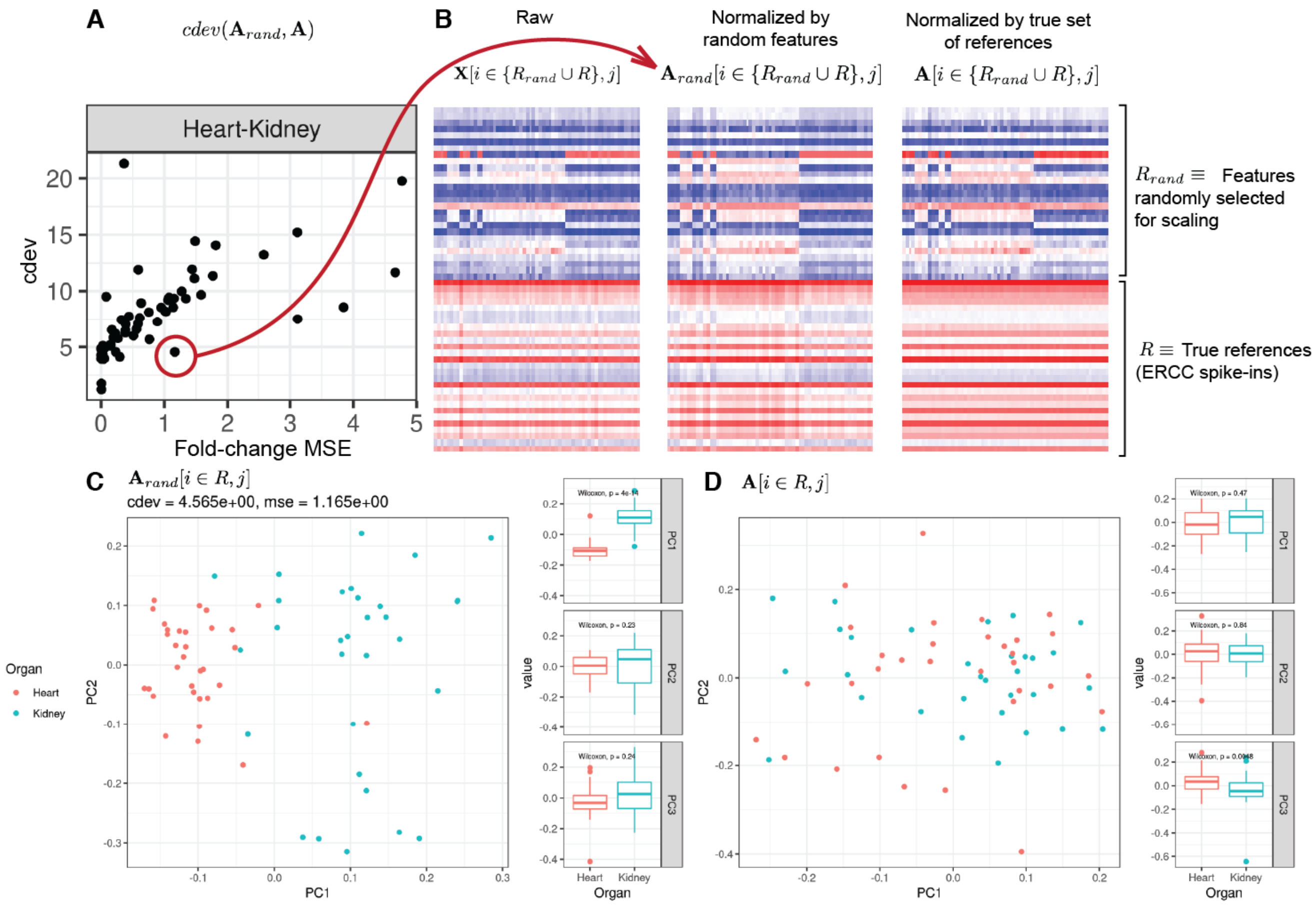

Fig. S7: **Additional close-up examination of discordance between** $cdev$ **and fold-change MSE on real data sets.** In labels of the plots, $\mathbf{A}$ denotes the ground truth obtained by normalizing against the true references R, $\mathbf{A}_{rand}$ the result of normalizing against a random set of genes R_{rand} which has the same number of genes as R, and $\mathbf{X}$ the un-normalized read count. In all cases, the column index j implies the selection of samples from heart and kidney.

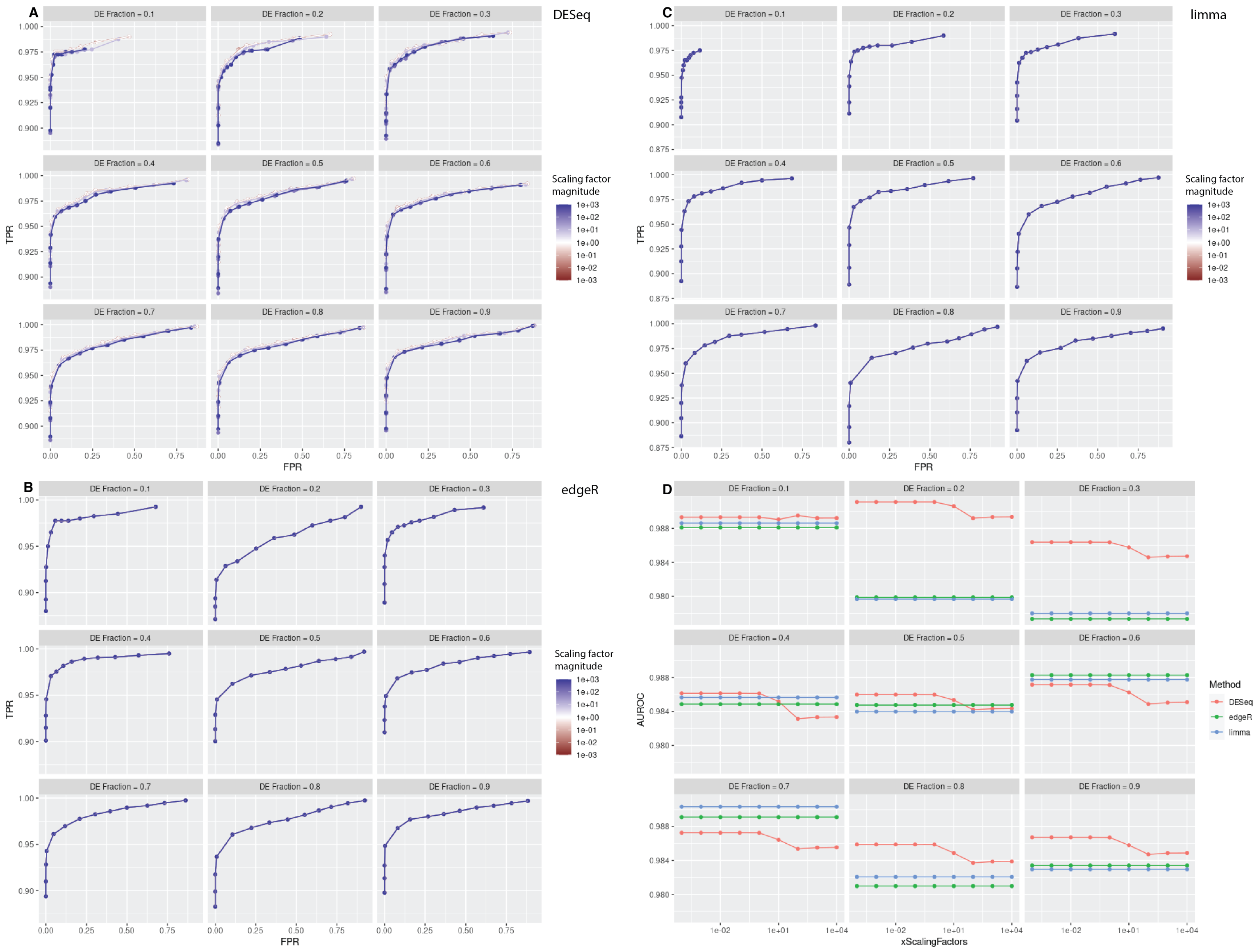

Fig. S8: **The effect of scaling magnitude on different DE detection methods**. The receiver operating characteristic (ROC) curve of DE detection using (**A**) DESeq, (**B**) edgeR, and (**C**) limma on simulation data normalized by multiples of the true scaling vector. (**D**) DE detection performance summarized by area under the ROC curve (AUROC). Note that the y-axis does not start from zero, to magnify the differences for better visibility.
